# Supplementary figures and images for: Rapid nucleus-scale reorganization of chromatin in neurons enables transcriptional adaptation for memory consolidation
Source: PLoS One. 2021 May 5;16(5):e0244038. doi: 10.1371/journal.pone.0244038 (PMC8099114; doi:10.1371/journal.pone.0244038)

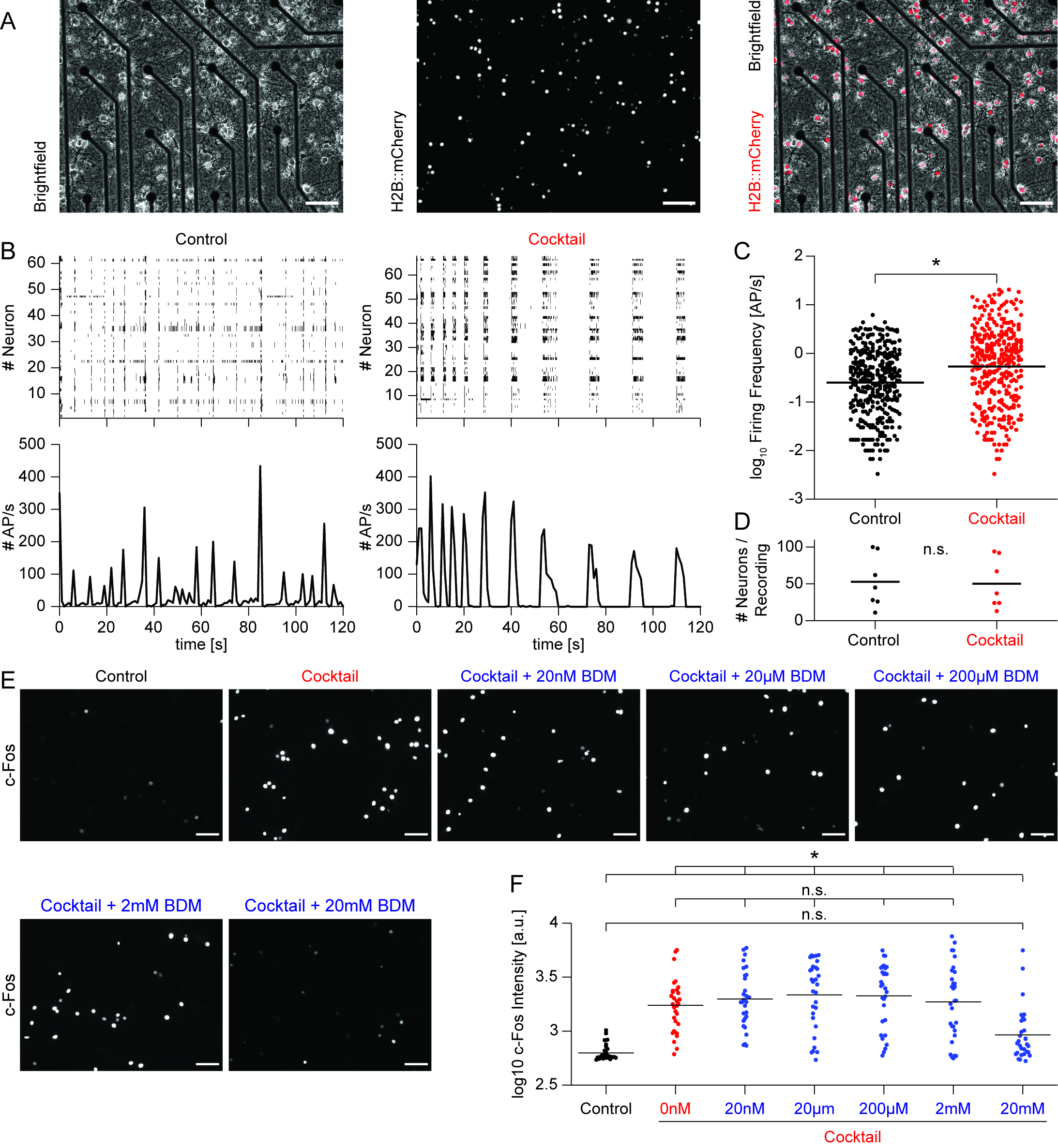

Supplement: S1 Fig — (A) Epifluorescence images of cultured primary neurons on a multielectrode array (MEA) with rAAV-mediated expression of H2B::mCherry. Left: Brightfield, middle: H2B::mCherry, right: Merged image; scale bar: 50 μm. (B) Top: Raster plots of single unit firing rates recorded on a MEA array under two different conditions. Bottom: Summed number of action potentials from all recorded single units in experiment shown on top. Left: Control, right: Cocktail. (C) Quantification of single unit firing frequency from all seven recordings (control, n = 370, mean±sem: 0.635±0.045; cocktail, n = 351, mean±sem: 1.99±0.19). Firing frequency is significantly increased during cocktail incubation (ANOVA, factor group, d.f.: 1, F = 36.63, p<0.001). (D) Comparable number of neurons per MEA recording (control, n = 7 recordings, mean±sem: 52.86±13.36; cocktail, n = 7 recordings, mean±sem: 50.14±12.80). The number of neurons per recording is not significantly different between groups (ANOVA, factor group, d.f.: 1, F = 0.01, p = 0.917). (E) Epifluorescence images of immunofluorescence staining against c-Fos after incubation of primary neurons with the pharmacological cocktail in combination with different concentrations of BDM. Respective condition is indicated on top of each image. (F) Quantification of c-Fos intensity for different conditions from panel (E) (control, n = 30, mean±sem: 636.68±22.32; cocktail, n = 30, mean±sem: 2027.02±230.43; cocktail+20nM, n = 30, mean±sem: 2362.70±267.47; cocktail+20μM, n = 30, mean±sem: 2692.40±282.05; cocktail+200μM, n = 30, mean±sem: 2582.23±264.83; cocktail+200μM, n = 30, mean±sem: 2497.58±351.20; cocktail+20mM, n = 30, mean±sem: 1133.77±193.57). c-Fos expression is not significantly different between control and cocktail+20mM BDM. All other conditions show significantly increased c-Fos expression (ANOVA, factor group, d.f.: 6, F = 18.41, p<0.001; differences between groups: Control vs. cocktail: p<0.001; control vs. cocktail+20nM: p<0.001; control vs. [file pone.0244038.s001.tif]

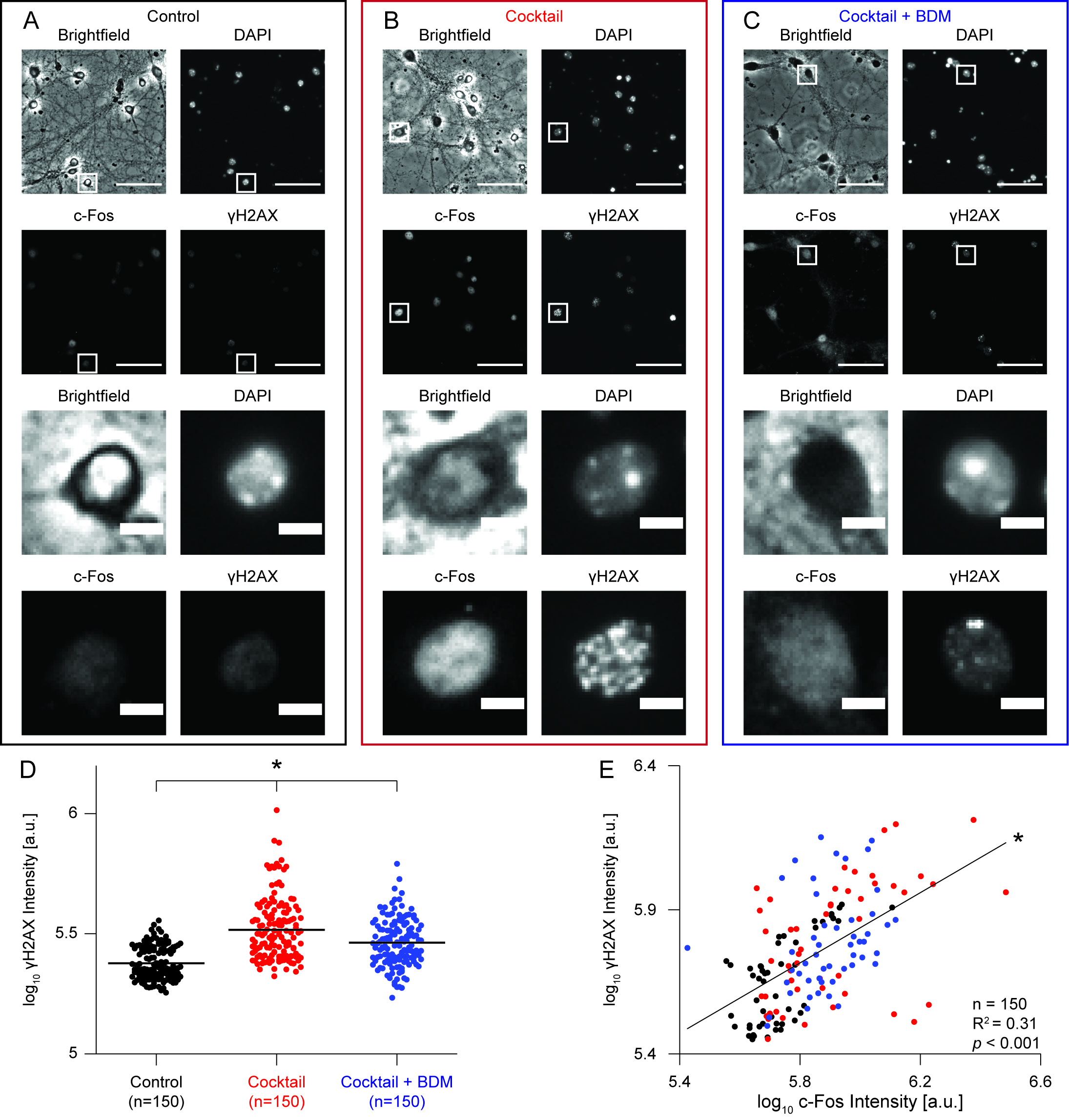

Supplement: S2 Fig — (A) Epifluorescence images of fixed cultured primary neurons from control condition after immunofluorescence staining for c-Fos and γH2AX, additionally stained with DAPI. Respective fluorescence channel is indicated on top of image. Scale bar: 50 μm. White squares indicate representative nucleus shown at higher magnification in bottom four images. Scale bar: 5 μm. (B) Same as panel (A) for cocktail condition. (C) Same as panel (A) for cocktail+BDM condition. (D) Dot plot of log10 γH2AX intensity for 150 individual cells from three different groups, respectively (control, black; cocktail, red; cocktail+BDM, blue; control: mean±sem: 301750±4991.2; cocktail: mean±sem: 477180±18360; cocktail+BDM: mean±sem: 394460±9659.3). γH2AX expression is significantly different between control and cocktail, control and cocktail+BDM, as well as between cocktail and cocktail+BDM (ANOVA, factor group, d.f.: 2, F = 73.26, p<0.001; differences between groups: Control vs. cocktail: p<0.001; control vs. cocktail+BDM: p<0.001; cocktail vs. cocktail+BDM: p<0.001). (E) Scatter plot of post-hoc log10 c-Fos intensity and log10 γH2AX intensity in 50 cells per group (n = 150 cells; black line: Linear fit, R2 = 0.31, p<0.001). Group color code is the same as in panel (D). (TIF) [file pone.0244038.s002.tif]

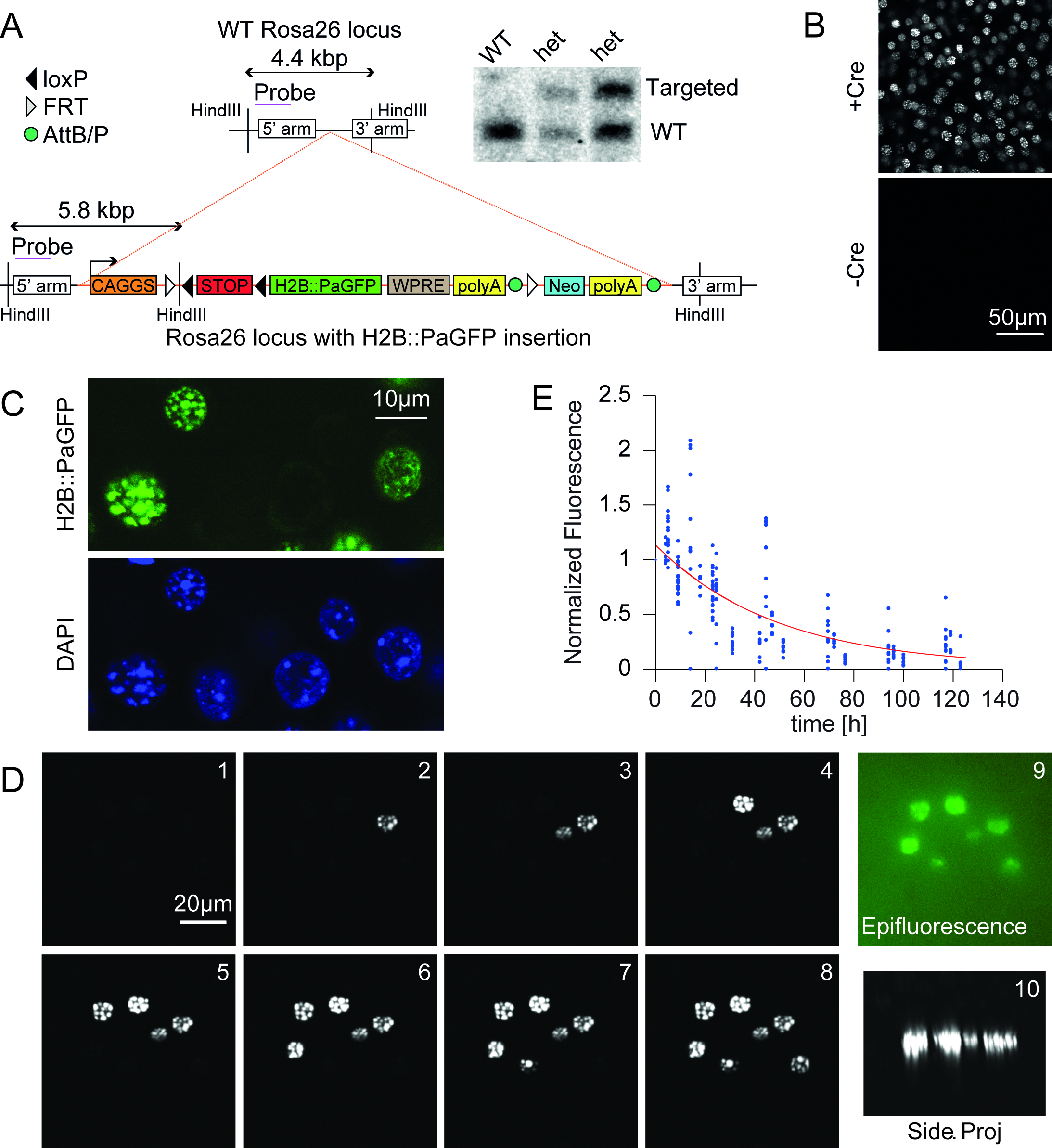

Supplement: S3 Fig — (A) Targeting strategy for the Rosa26 locus and southern blots of wild-type and H2B::PaGFP heterozygous mice. H2B::PaGFP is expressed from the CAGGS promoter, directly upstream of the transgene is a floxed STOP cassette. (B) Top panel: Confocal image of a fixed section of the auditory cortex from a H2B::PaGFP mouse crossed with an EMX1-Cre mouse line after broad photolabeling. Bottom panel: Analog image from a Cre negative H2B::PaGFP littermate. (C) Confocal image of a fixed section of the auditory cortex taken from a H2B::PaGFP mouse with rAAV-mediated Cre expression. Top panel: H2B::PaGFP fluorescence after broad photolabeling in the imaging plane, bottom panel shows DAPI fluorescence. A strong correlation between both signals can be observed in transduced cells. (D) Panel 1–8: Maximum intensity projection from in vivo two-photon image stacks of consecutively photolabeled nuclei in the auditory cortex. Note selective activation of H2B::PaGFP fluorescence in nuclei which have been photoactivated at 750 nm. Panel 9: In vivo epifluorescence image of the same activated nuclei. Panel 10: Side projection of an image stack of the activated nuclei from panel 8, illustrating selective photoactivation in the imaging plane. (TIF) [file pone.0244038.s003.tif]

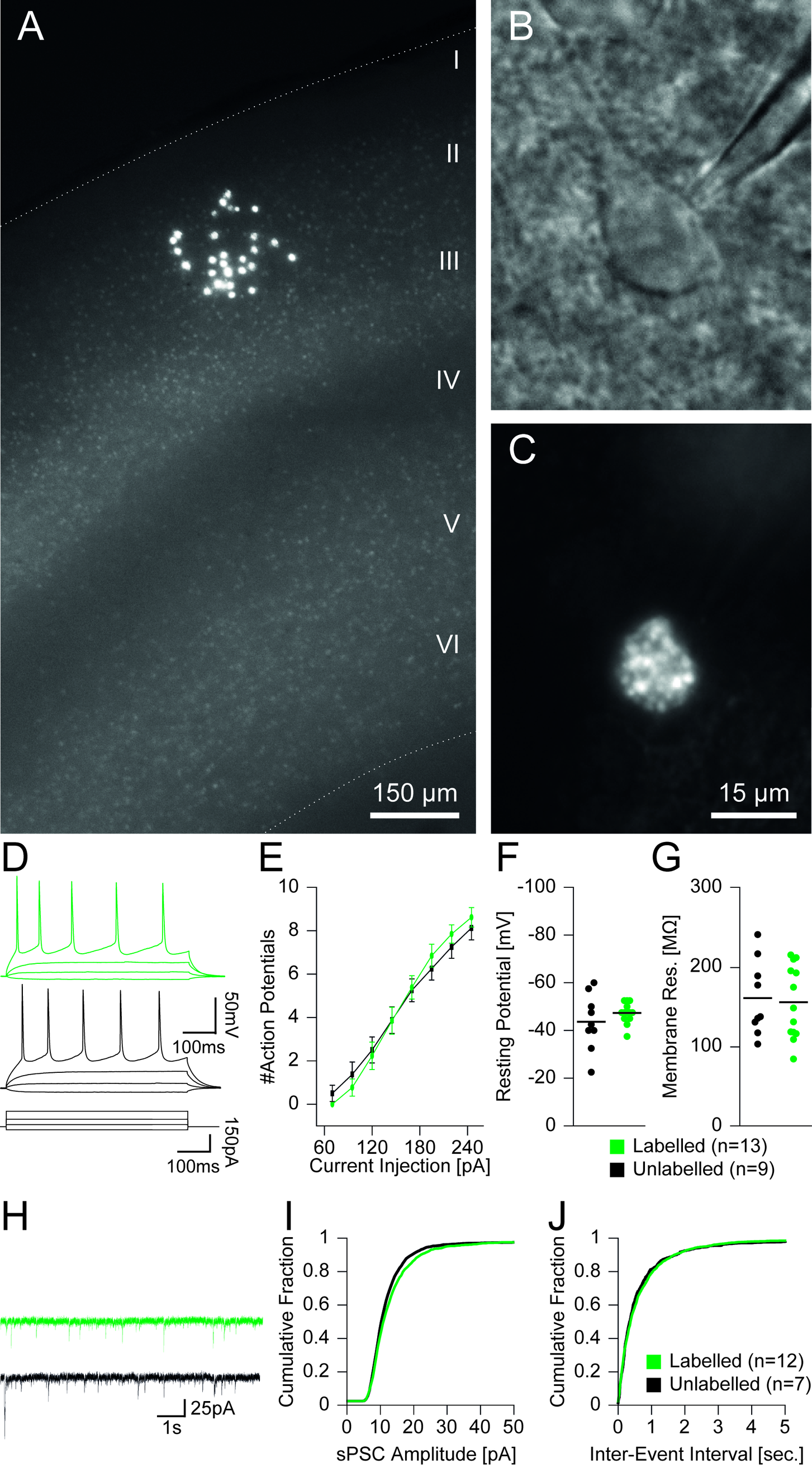

Supplement: S4 Fig — (A) Epifluorescence image of an acute coronal brain slice of a H2B::Pa-GFP mouse crossed with a CaMKIIa-Cre mouse with a field of two-photon photolabeled cells in layers II/III. (B) Dodt-contrast image of the soma of a pyramidal neuron with a patch-pipette in an acute brain slice. (C) Corresponding epifluorescence image of panel (B) showing strong photolabel of the neuronal chromatin. (D) Patch-clamp whole-cell current-clamp recordings of a photolabeled (green) and non-labeled (black) nearby neuron in response to current injections of various amplitudes. (E) Input/output relationship of injected current and elicited action potentials in photolabeled (green) and non-labeled neurons (black). Photolabeled neurons: n = 13; 20pA: 0±0.0 AP; 45pA: 0±0.0 AP; 70pA: 0±0.1 AP; 95pA: 0±0.4 AP; 120pA: 1±0.6 AP; 145pA: 4±0.6 AP; 170pA: 5±0.5 AP; 195pA: 7±0.5 AP; 220pA: 8±0.4 AP; 245pA 9±0.4 AP; Non-labeled neurons; n = 8; 20pA: 0±0.0 AP; 45pA: 0±0.0 AP; 70pA: 0±0.4 AP; 95pA: 1±0.6 AP; 120pA: 2±0.6 AP; 145pA: 4±0.6 AP; 170pA: 5±0.5 AP; 195pA: 6±0.5 AP; 220pA: 7±0.5 AP; 245pA 8±0.5 AP; all values are median±sem; Two-way ANOVA: Significant effect of current injection (d.f.: 9, F = 211, p<0.001), no significant effect of photolabeling treatment (d.f.: 1, F = 0.009, p = 0.93) or interaction (d.f.:9, F = 0.91, p = 0.53); (F) Resting potential in photolabeled (green) and non-labeled neurons (black). Photolabeled neurons: n = 13; -79±0.5 mV; Non-labeled neurons: n = 9; -77±1.6 mV; p = 0.40, Mann-Whitney test. (G) Membrane resistance in photolabeled (green) and non-labeled neurons (black). Photolabeled neurons: n = 13; 148.2±12.7 MΩ; Non-labeled neurons: n = 9; 137.3±15.8 MΩ; p = 0.79, Mann-Whitney test. (H) Voltage-clamp registration at -70mV holding potential of spontaneous postsynaptic currents (sPSCs) in photolabeled (green) and non-labeled neurons (black). Photolabeled neurons: n = 2021 events, 12 cells; 10.58±0.1 pA; Non-labeled neurons: n = 1146 events, 7 cells; 11.23±0.2 pA; p = 0 [file pone.0244038.s004.tif]

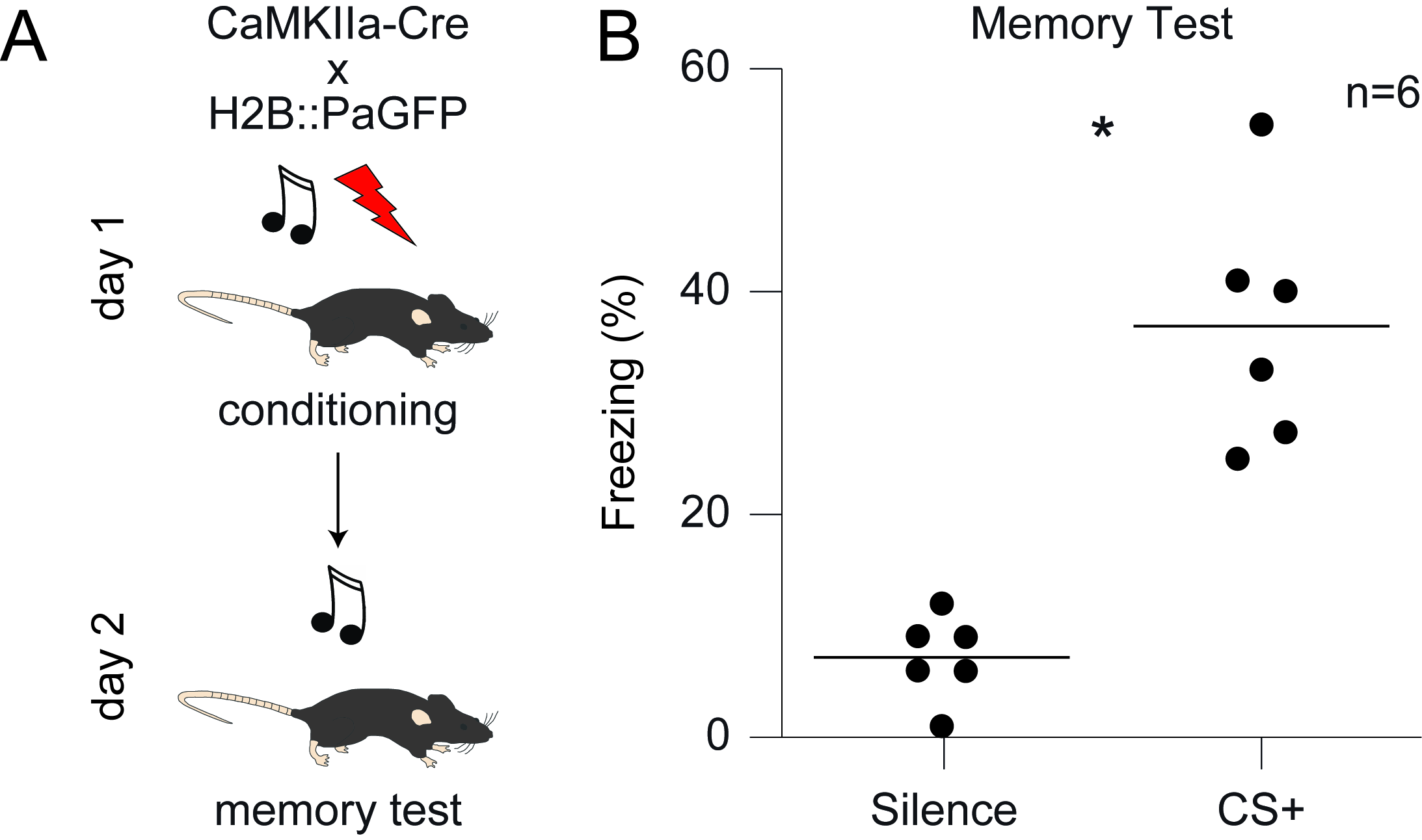

Supplement: S5 Fig — (A) Offspring of a cross of CaMKIIa-Cre and H2B::Pa-GFP mice underwent auditory cued fear conditioning following a standard protocol involving five sound/foot shock pairings to a complex sound CS+ (see Materials and methods). (B) Quantification of the memory test session 24 hours after auditory cued fear conditioning. Mice showed increased freezing upon CS+ presentation, indicating successful formation of an associative memory (n = 6, Silence: 0.072±0.015, CS+: 0.369±0.045; Wilcoxon rank sum test, p<0.003). (TIF) [file pone.0244038.s005.tif]

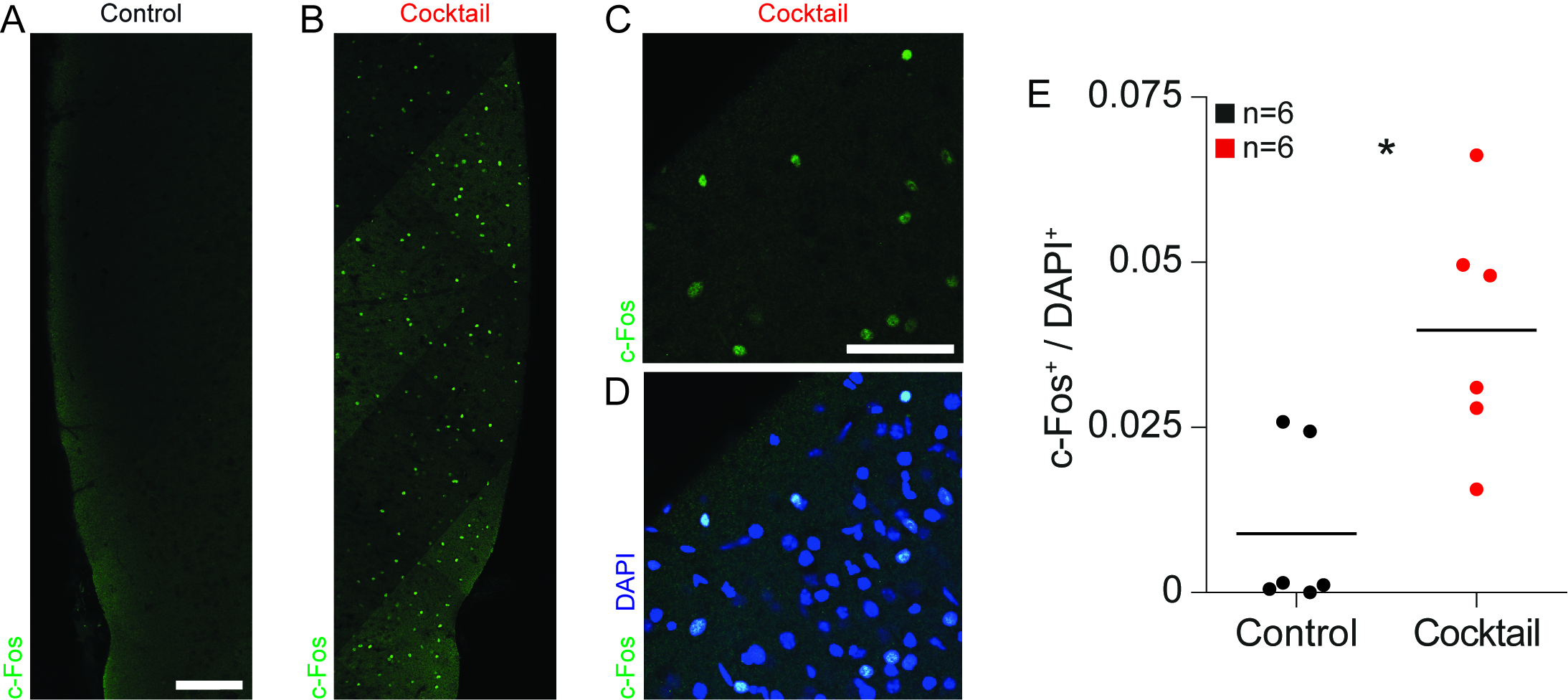

Supplement: S6 Fig — (A) Confocal image of a control brain slice fixed 1 hour after sham treatment and subsequent immunofluorescence detection of the IEG product c-Fos. Scale bar: 150 μm. (B) Analog image as shown in panel (A), however, brain slice was treated for 1 hour with cocktail. Note increase in the number of c-Fos-positive neurons. (C) Higher magnification image of c-Fos-positive cells in a brain slices incubated with cocktail. Scale bar: 50 μm. (D) Same image, additionally DAPI staining of nuclei shown in blue. (E) Quantification of c-Fos-positive neurons in control brain slices incubated with ACSF (black) and brain slices additionally incubated with cocktail (red). Control: n = 6; 0.13±0.51%; Cocktail: n = 6; 3.95±0.74%; all values are median±sem. Mann-Whitney test: p<0.01. (TIF) [file pone.0244038.s006.tif]

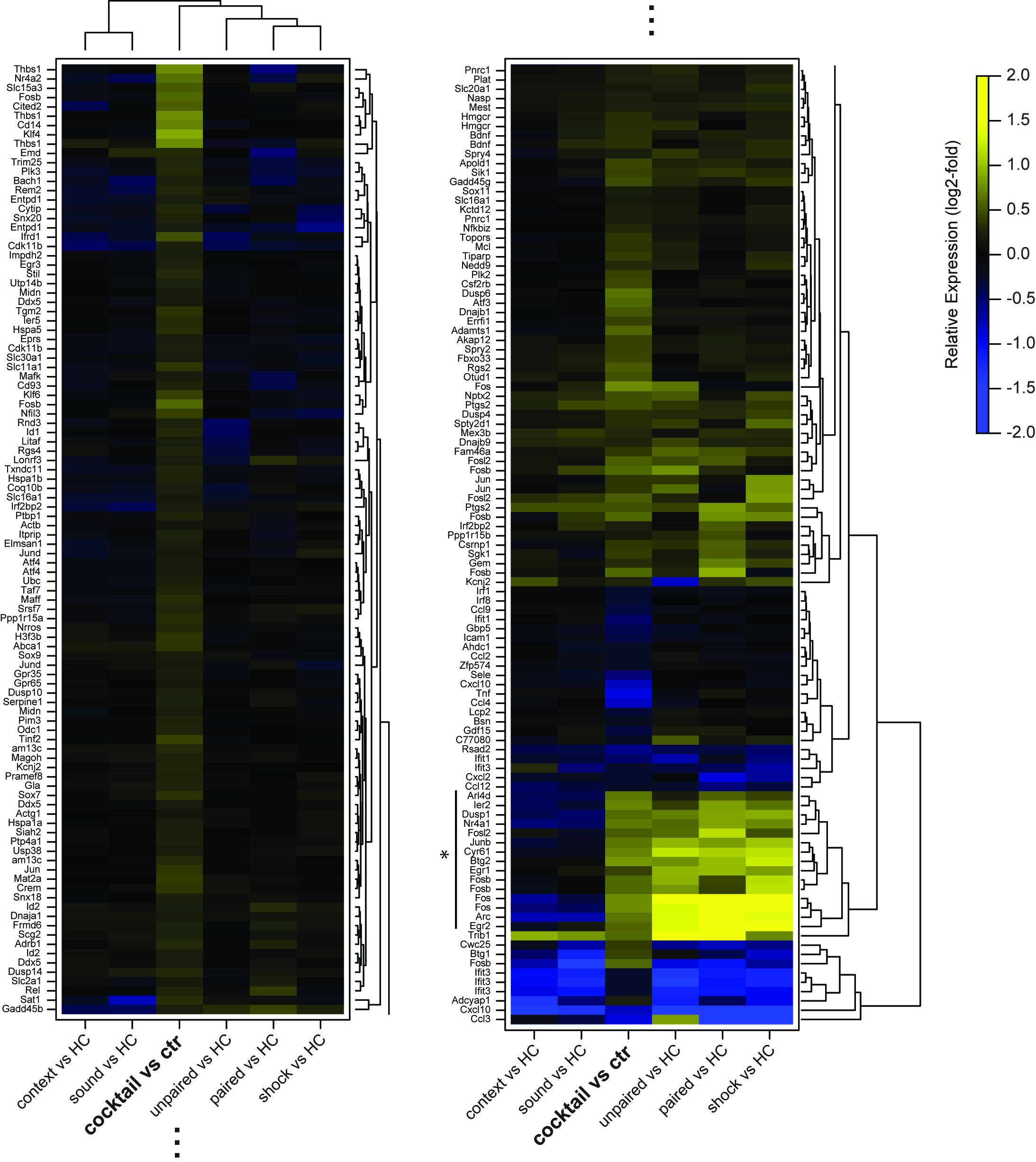

Supplement: S7 Fig — Heatmap display showing significantly differentially expressed genes analyzed by RNAseq in six acute brain slices treated with the pharmacological cocktail and six control slices (column ‘cocktail vs. control’). In a previous study, we analyzed the effect of various behavioral treatments (paired auditory cued fear conditioning, unpaired auditory cued fear conditioning, shock presentation only, sound presentation only, temporary housing in neutral context, housing in home cage) on gene expression using a microarray-based analysis. Here, we reported, considering the relative expression levels of individual genes, that many known immediate-early genes were induced in those mice undergoing a treatment involving a shock [34]; EMBL-EBI ArrayExpress, Accession number: E-MTAB-661). For comparison, corresponding relative expression levels from this in vivo study are displayed as additional columns. Expression levels of some genes were measured with several probes on the microarray and are therefore mentioned multiple times. Fig 3E of the main text of the manuscript shows a subcluster of the heatmap display containing many known immediate-early genes. (TIF) [file pone.0244038.s007.tif]

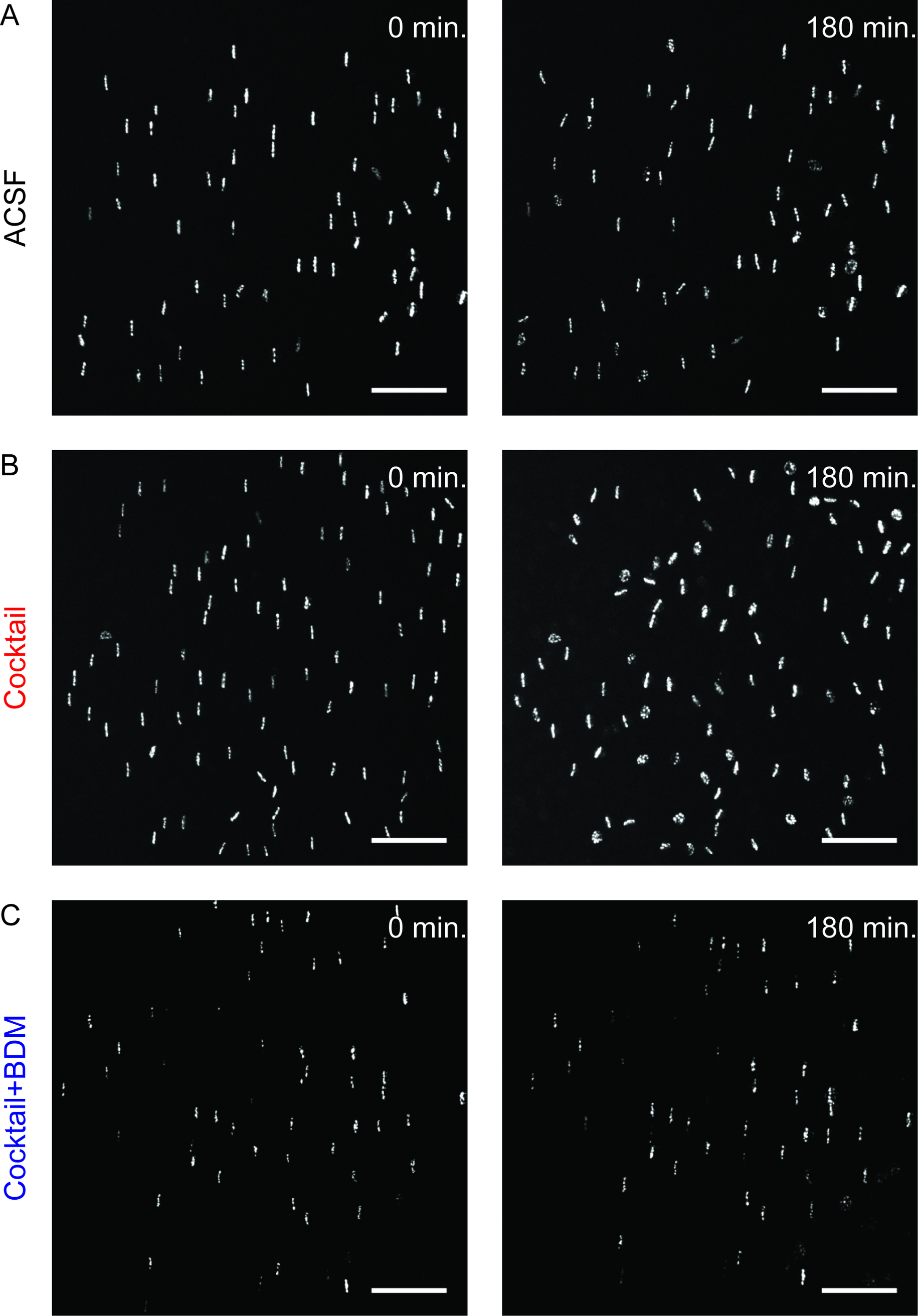

Supplement: S8 Fig — (A) Maximum intensity projection of an experiment with mock manipulation using ACSF. Left: image acquired directly after photolabeling. Right: Image acquired 180 minutes after photolabeling. (B) Analog to panel (A), but for a slice which was manipulated by exchange of the perfusion solution to the pharmacological cocktail between 60 and 120 minutes after photolabeling. (C) Analog to panel (A), but for a slice which was treated with the pharmacological cocktail and BDM. (TIF) [file pone.0244038.s008.tif]

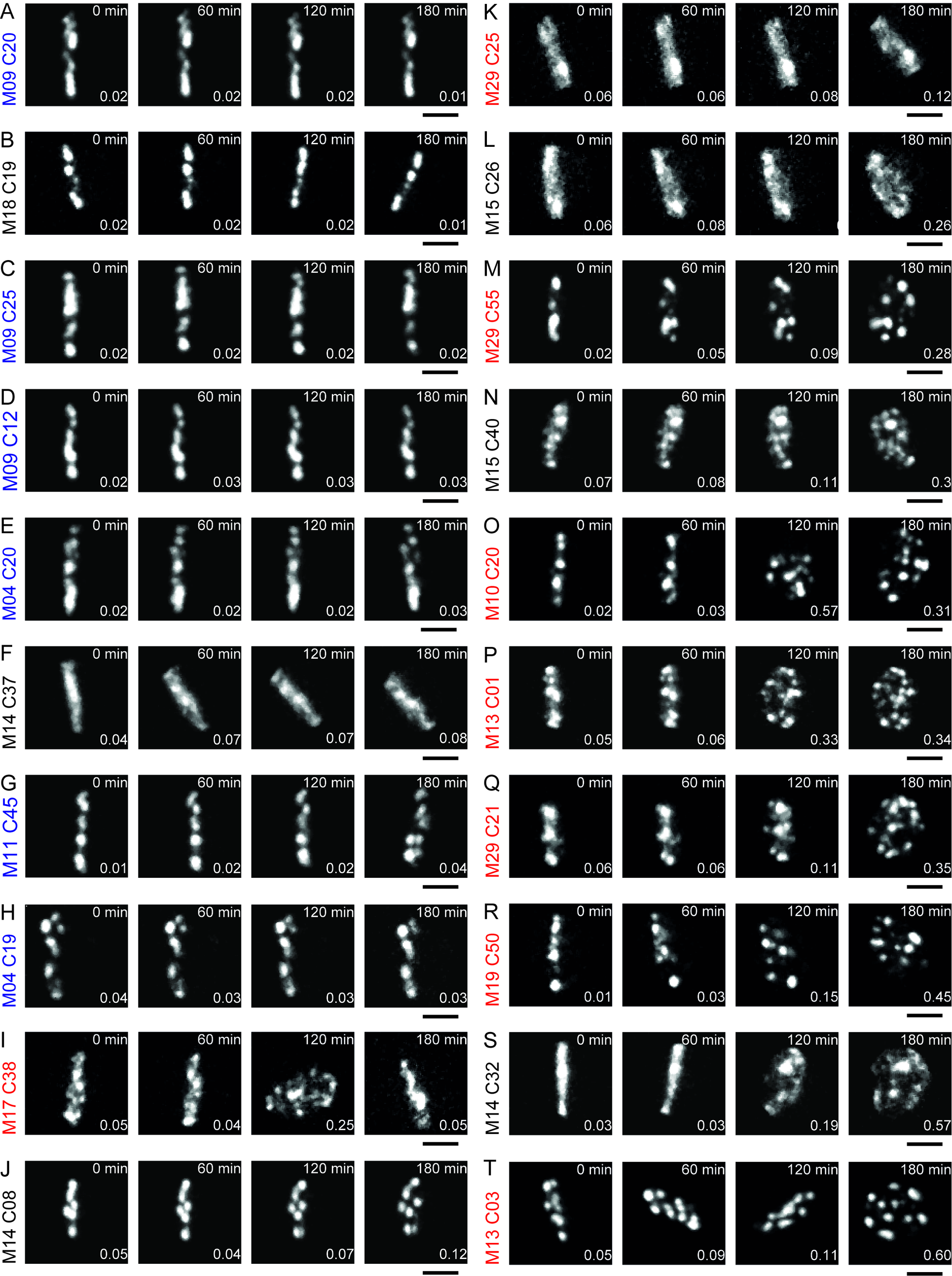

Supplement: S9 Fig — Despite clear overall effects between the different treatment conditions (Fig 3 of the main manuscript), we observed in acute brain slices substantial heterogeneity across individual nuclei within a treatment group. (A) to (T): Maximum intensity projections of two-photon image stacks of 20 cells from experiments observing chromatin dynamics in slices from H2B::Pa-GFP crossed with CaMKIIa-Cre mice from all three treatment conditions illustrating heterogeneous chromatin dynamics. Text left of image series depicts identity of cell and slice (Black: Control; Red: Cocktail; Blue: Cockatil+BDM; scale bar = 5 μm). (TIF) [file pone.0244038.s009.tif]

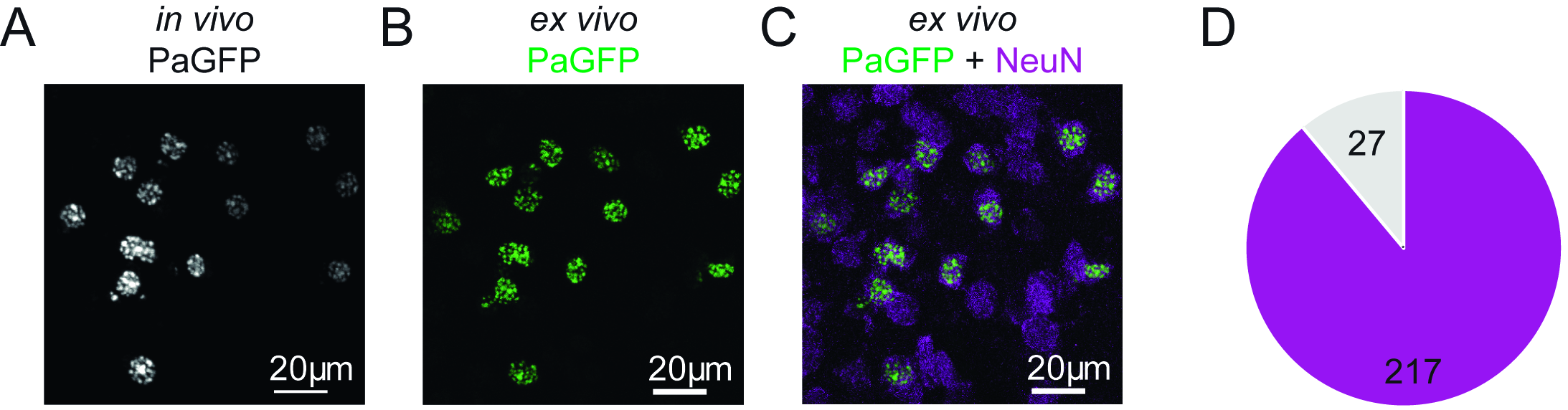

Supplement: S10 Fig — (A) Maximum intensity projection of a in vivo two-photon image stack of photolabeled cells in layers II/III of the mouse auditory cortex of a H2B::Pa-GFP mouse with rAAV-mediated Cre-expression under the control of the CMV promoter. We selected for large nuclei for photolabeling that could be identified prior labeling using higher laser intensities at 900nm due to basal fluorescence of Pa-GFP. (B) Maximum intensity projection of a confocal image stack of the same cells after fixation and tangential slicing of the brain. Strong labeling of the cells readily enables re-identification. (C) Maximum intensity projection of the same stack merged with a second imaging channel for spectrally separated immunofluorescence staining against the neuronal marker protein NeuN in magenta. Most labeled cells show co-staining for NeuN. (D) Quantification of the immunofluorescence analysis: Out of a total of 244 in vivo photolabeled cells from 7 mice 217 were NeuN positive. The effective transduction of neurons by rAAV5 [85] and the selection of large nuclei for photolabeling are factors contributing to the strong bias towards labeling neurons. (TIF) [file pone.0244038.s010.tif]

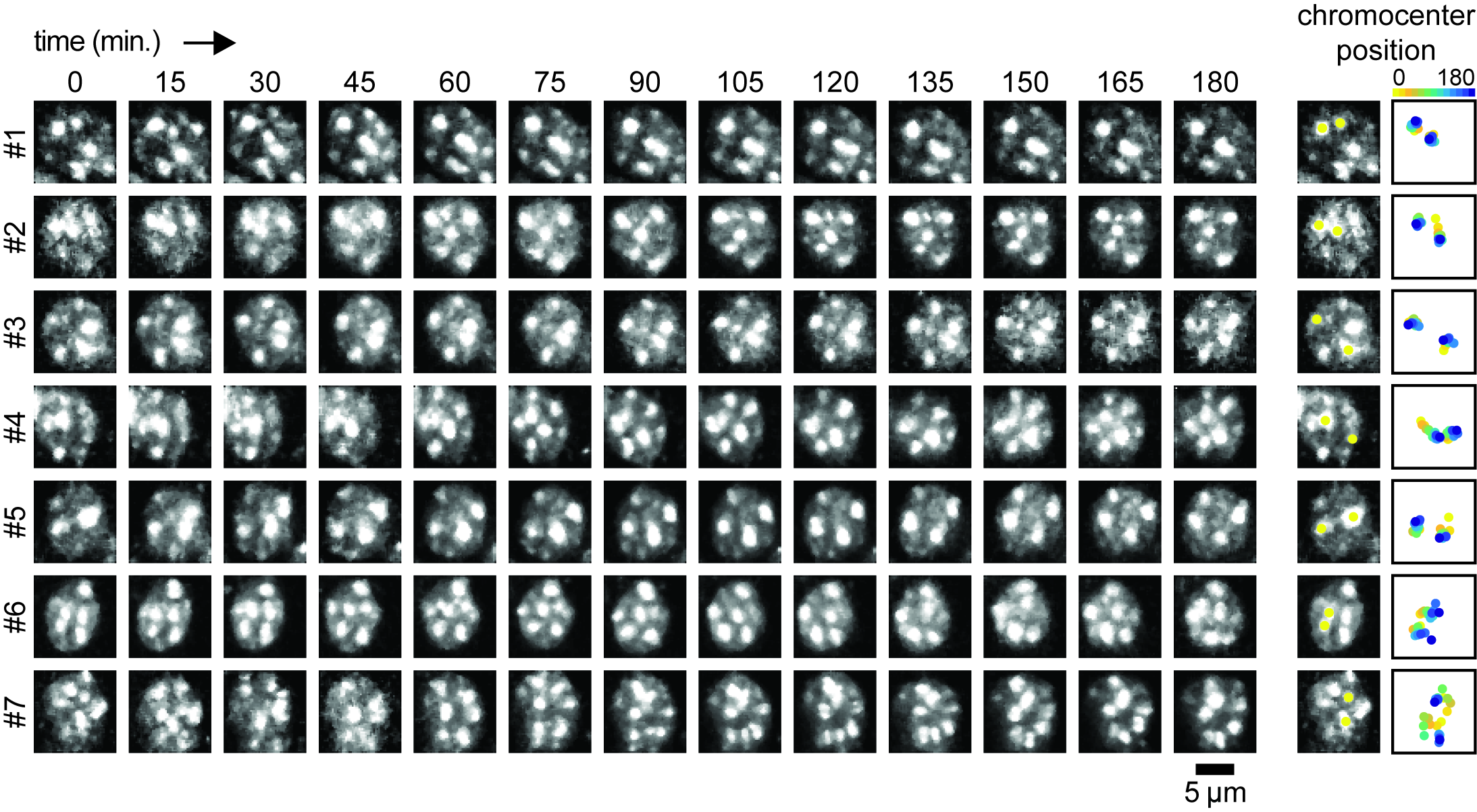

Supplement: S11 Fig — (A) Maximum intensity projections of a deconvolved confocal image stack taken from an individual neuron photolabeled 1 hour before fixation. (B) Same as panel (A) from an individual neuron photolabeled 18 hours before fixation. Different projections illustrate the spread of the photolabeled subset of chromatin along all three spatial dimensions. (C) Three eigenvalues estimated in deconvolved confocal image stacks from neurons fixed 1 hour (red) or 18 hours (blue) after photolabeling. The second and third eigenvalues increase in cells fixed 18 hours after photolabeling indicating spread of the label in all three dimensions. (D) Correlation of the CI calculated using the maximum intensity projection along the z-axis and the third eigenvalue estimated in the analysis considering all three dimensions in neurons fixed 1 hour (red) and 18 hours (blue) after photolabeling. In cultured cells, brain slices as well as in vivo, we observed rotational movements of the chromatin in many cells. This became particularly obvious in cells in which the photolabeled line was preserved but rotated. This phenotype suggests that nuclear chromatin can move entirely as a rigid body without changing its internal architecture. To test if increases in CI values describing a loss of the line structure are associated with a three-dimensional redistribution of the photolabeled chromatin throughout the nucleus, we performed in vivo photolabeling of neurons 18 and 1 hour before fixation, leading to two groups of cells that will, on average, have undergone more or less spontaneous reorganization of chromatin. The photolabeled cells were re-identified in the fixed tissue and imaged using confocal microscopy and subsequent image deconvolution to improve the z resolution of the images. We analyzed the three-dimensional spread of the fluorescence in the image stack using a principal component analysis. Rigid rotational movement would leave the pattern of the photolabel unaltered and, therefore would no [file pone.0244038.s011.tif]

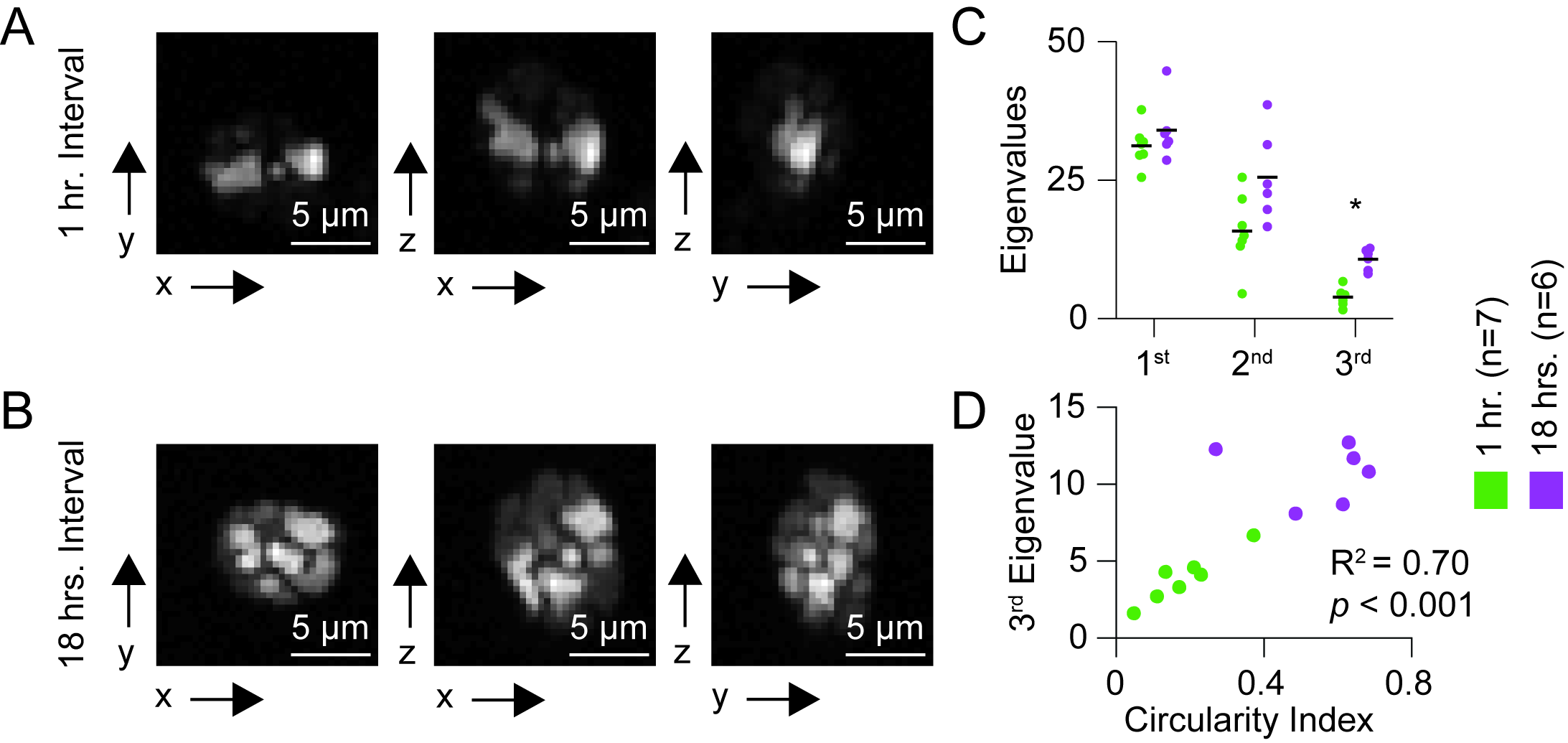

Supplement: S12 Fig — Wild-type C57/Bl6 mice received stereotaxic injections of Hoechst and subsequently a cranial widow was implanted over the auditory cortex. Time-lapse two-photon images (maximum intensity projections) taken from individual nuclei at a 15 min interval during isoflurane anesthesia. Right panels show tracking of the position of two chromocenters per cell over the imaging period with time color coded. Note that, similar to the observations obtained with photolabeling of nucleosomes, different cells show varying levels of chromatin dynamics and phases of relative stability can be interrupted by phases of increased motility. (TIF) [file pone.0244038.s012.tif]

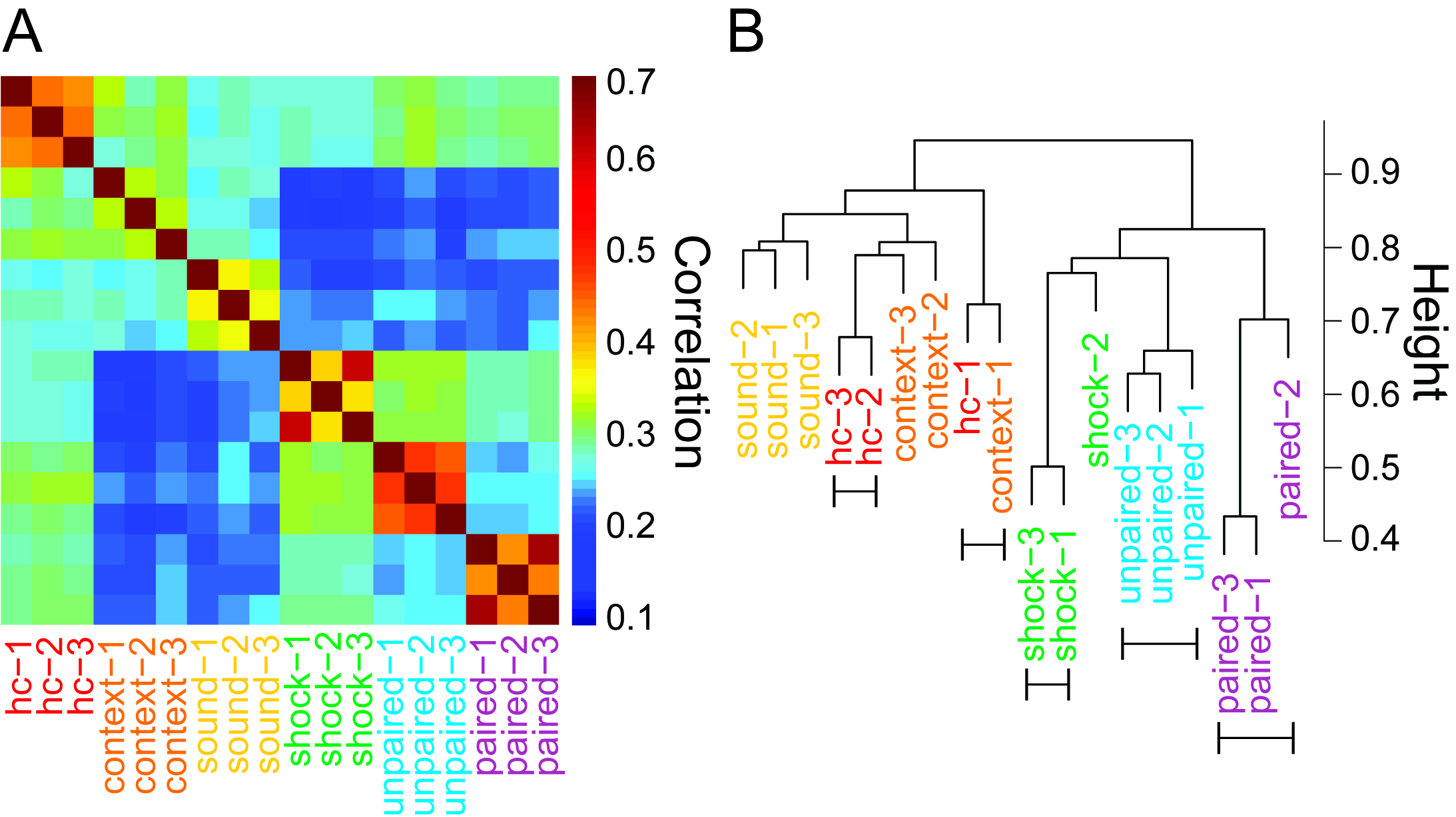

Supplement: S13 Fig — A re-analysis of the data from the previous microarray study [34] taking into account the expression pattern of all genes on the microarray revealed that the biological replicates of mice undergoing either paired or unpaired conditioned could be identified based on the analysis of gene expression patterns. (A) Spearman correlation plot of the complete expression profiles, three independent biological replicates per treatment. Paired and unpaired conditioning and to some extend shock presentation only result in a good correlation of their overall expression profile within replicates but is particular distinct from each other as well as from sound presentation and housing in a neutral context. (B) Dendrogram of a cluster analysis of the complete expression profiles. The uncertainty in clustering was assessed with the multiscale bootstrap resampling of pvclust (nboot = 10000). The bars below indicate clusters with an AU p-value > 0.95. (TIF) [file pone.0244038.s013.tif]

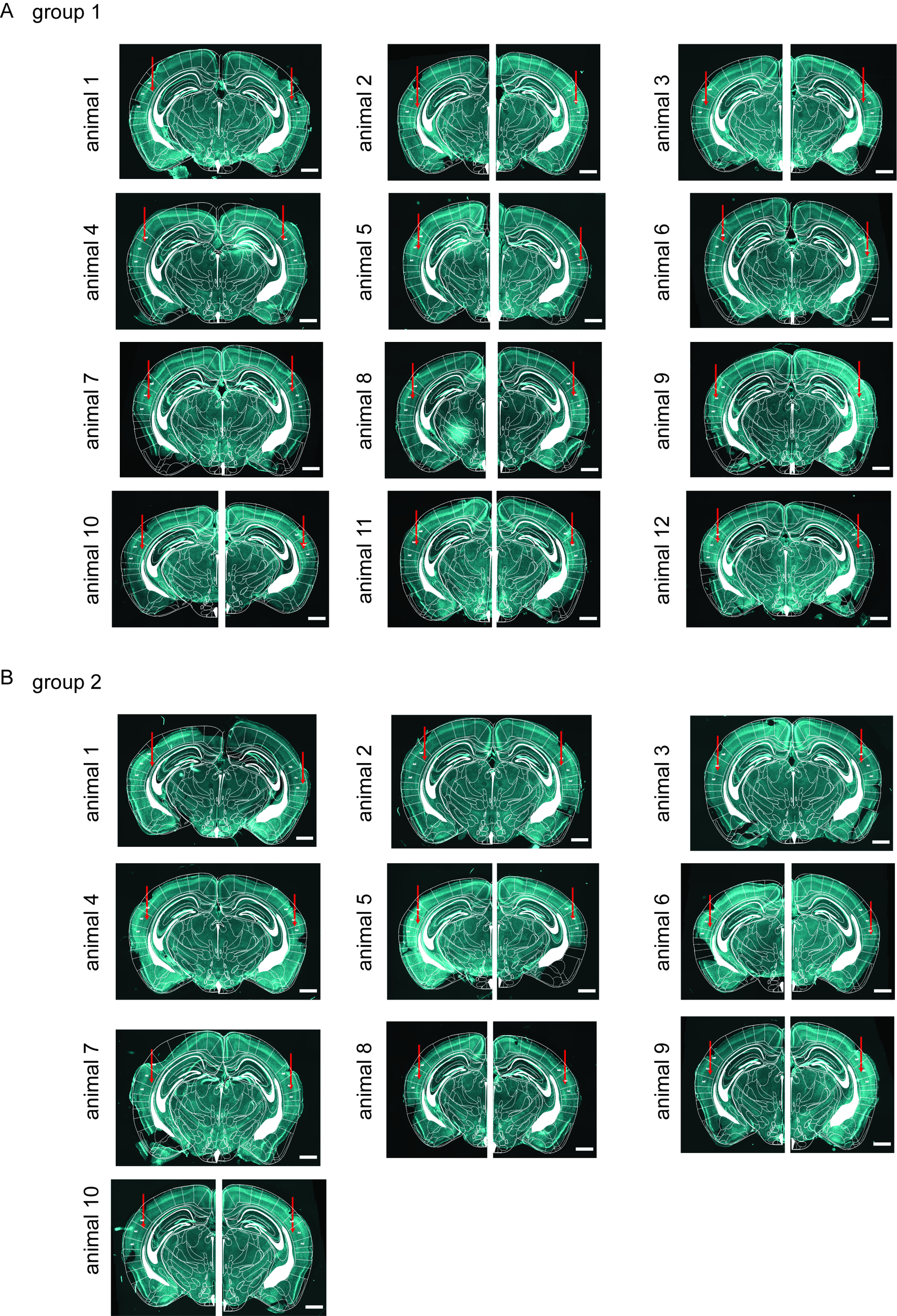

Supplement: S14 Fig — (A) Epifluorescence images of DAPI-stained coronal brain sections of mice from group 1 of behavioral experiment to study the impact of bilateral BDM infusion into the auditory cortex. Transparent overlay is from the corresponding section of a mouse brain atlas. Red arrow depicts position of cannula (scale bar = 1 mm). (B) Same as panel (A) for group 2 of behavioral experiment. (TIF) [file pone.0244038.s014.tif]

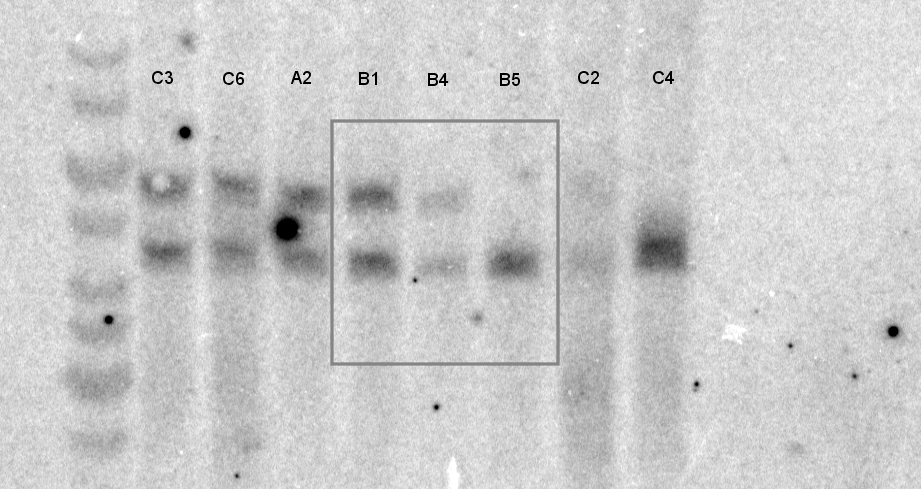

Supplement: S1 File — (TIF) [file pone.0244038.s018.tif]

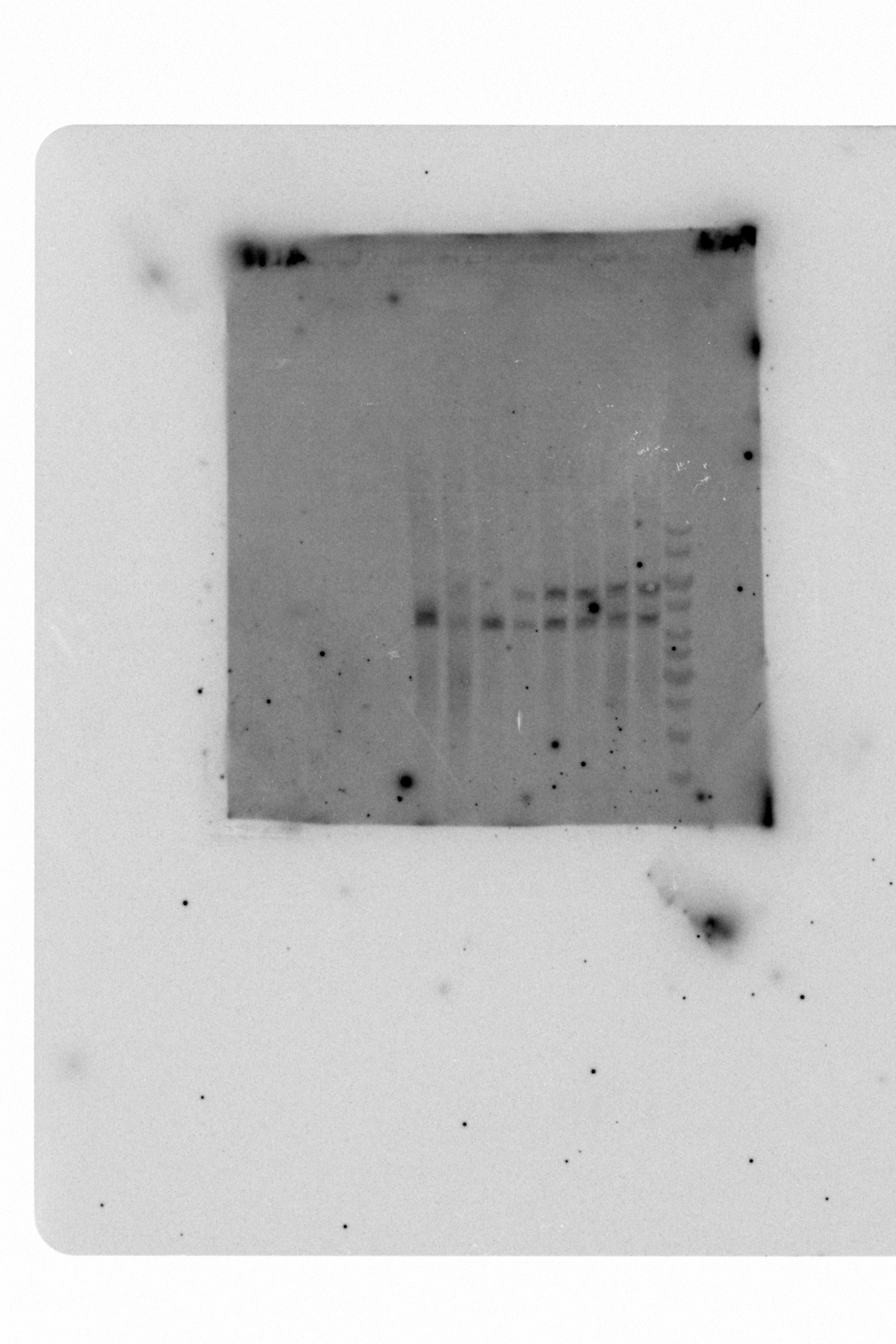

Supplement: S2 File — (GEL) [file pone.0244038.s019.gel]
